# Midline Angle as a Predictor of Neonatal Orbital Compression During Forceps Delivery in Occiput Anterior Presentations

**DOI:** 10.1111/jog.70520

**Published:** 2026-10-26

**Authors:** Seisuke Sayama, Takahiro Seyama, Takayuki Iriyama, Eriko Yano, Haruka Matsui, Kensuke Suzuki, Mari Ichinose, Masatake Toshimitsu, Keiichi Kumasawa, Miyuki Harada, Yasushi Hirota

**Affiliations:** ^1^ Department of Obstetrics and Gynecology, Graduate School of Medicine The University of Tokyo Tokyo Japan; ^2^ Department of Obstetrics and Gynecology, Graduate School of Medicine Teikyo University Tokyo Japan

**Keywords:** forceps delivery, intrapartum ultrasound, midline angle, neonatal trauma, orbital injury

## Abstract

**Aim:**

To evaluate the association between intrapartum transperineal ultrasonography findings obtained before forceps delivery and neonatal periorbital compression marks in occiput anterior presentations.

**Methods:**

This retrospective observational study included 166 term singleton forceps deliveries performed at the University of Tokyo Hospital between January 2019 and December 2022. Pregnancies with a non‐cephalic presentation, congenital anomalies, or occiput posterior or transverse positions were excluded. Maternal and neonatal characteristics, together with intrapartum transperineal ultrasound findings, including the angle of progression and the midline angle retrospectively measured from ultrasound images acquired immediately before forceps delivery, were compared between neonates with periorbital forceps compression marks and those without compression marks. All neonates in the compression mark group underwent ophthalmologic evaluation.

**Results:**

The median midline angle was significantly greater in the compression mark group than in the control group (22.0° vs. 13.0°, *p* < 0.001). No significant differences were observed between the two groups in maternal or neonatal characteristics or in the angle of progression. The cutoff value of 22° yielded a sensitivity of 47.8% and a specificity of 90.9% for predicting forceps‐related compression marks. Nine of the 67 neonates with compression marks had confirmed ophthalmic findings: retinal hemorrhage (*n* = 1), corneal epithelial injury (*n* = 2), and subconjunctival hemorrhage (*n* = 6). All findings resolved spontaneously without intervention.

**Conclusions:**

A midline angle of > 22° was associated with an increased likelihood of periorbital compression during forceps delivery in occiput anterior presentations. Midline angle assessment may be a useful adjunctive parameter for assessing the risk of periorbital compression marks.

## 1 Introduction

Forceps delivery remains an important obstetric intervention for expediting birth during the second stage of labor when appropriately indicated and properly performed. However, it is associated with specific neonatal risks, including ocular and orbital injuries. Although uncommon, these injuries may result in substantial morbidity, ranging from transient corneal epithelial injury to more severe complications, such as corneal laceration, Descemet's membrane rupture, and in rare cases, globe rupture or avulsion, which may lead to permanent corneal opacity and long‐term visual impairment [1, 2].

Retrospective and prospective studies have reported the incidence of ocular injury after forceps delivery [2, 3]. A recent prospective cohort study identified ophthalmological abnormalities, including corneal edema, retinal hemorrhage, and subconjunctival hemorrhage, in up to 17% of neonates delivered by forceps [4]. Notably, fetal head malrotation has been implicated as a contributing factor in the pathophysiology of such injuries, especially in the transverse and occiput posterior positions [4]. However, these injuries may also occur in occiput anterior presentations with mild obliquity, such as right or left occiput anterior positions, although the degree of obliquity associated with injury remains unclear [5, 6].

Accurate determination of fetal head position is critical when planning operative vaginal delivery. Traditional digital examination is often hindered by caput succedaneum and molding, leading to misclassification of fetal head position in up to 30% of cases [7]. Intrapartum transperineal ultrasound (TPU) has emerged as a valuable adjunct for the real‐time, objective assessment of fetal head position, station, and rotation [8]. While the angle of progression (AoP) is a sonographic parameter of fetal head descent, the midline angle (MA), defined as the angle between the fetal cranial midline and the maternal anteroposterior pelvic axis in the axial plane, provides a direct measure of fetal head rotation [9]. Because MA reflects fetal head rotation, it may provide information about the orientation of the fetal orbit relative to the forceps blades. However, to the best of our knowledge, no prior studies have specifically evaluated the association between MA values and neonatal periorbital compression marks following forceps delivery. Clinically significant ophthalmic injuries are rare; however, externally visible periorbital compression marks occur more frequently and may indicate contact between the forceps blade and the periorbital region [10]. These marks do not themselves constitute ophthalmic injury but may provide a practical surrogate outcome for investigating the mechanical effects of forceps application [5, 11, 12].

The objective of the present study was to evaluate the association between MA and AoP measured prior to forceps delivery with neonatal periorbital compression marks in occiput anterior presentation. Periorbital compression marks were evaluated as a surrogate indicator of forceps blade compression over the periorbital region and were not considered equivalent to clinically confirmed ophthalmic injury. Ophthalmologist‐confirmed findings among neonates with compression marks were evaluated descriptively as a secondary outcome.

## 2 Methods

This retrospective observational study was conducted at the University of Tokyo Hospital from January 2019 to December 2022. The study protocol was approved by the Institutional Review Board of the University of Tokyo (approval number, 3053). In accordance with the institutional policy and retrospective design, the requirement for individual informed consent was waived, and an opt‐out option was provided through the hospital website. Clinical information was retrieved from the medical records of women with live singleton fetuses in cephalic presentations at the University of Tokyo Hospital between January 2019 and December 2022. No artificial intelligence tools, including large language models such as ChatGPT, were used in the preparation of this manuscript.

### 2.1 Study Population

We included pregnant women who underwent term forceps delivery (at ≥ 37 + 0 weeks and ≤ 41 + 6 weeks) with singleton fetuses in occiput anterior presentation using UTokyo Naegele Forceps. Indications for forceps delivery included labor arrest, non‐reassuring fetal status, and maternal conditions such as preeclampsia. The exclusion criteria were (1) non‐cephalic presentation, (2) known congenital anomalies, and (3) occiput posterior or transverse presentation, as assessed by intrapartum ultrasound, as previously described [9]. In accordance with our institutional policy, forceps delivery was performed when the MA was < 45° and the fetal head station was at or beyond +3. All consecutive eligible forceps deliveries performed between January 2019 and December 2022 were included. Neonates with periorbital compression marks were classified as the compression mark group, whereas those without such marks were classified as the control group.

### 2.2 Ultrasound Acquisition and Measurement of Midline Angle and Angle of Progression

TPU was performed using a Voluson P8 system (GE Healthcare, Chicago, IL, USA) equipped with a 2‐ to 5‐MHz convex transducer. Ultrasound examinations were performed immediately before forceps application, with the patient in a semi‐recumbent lithotomy position, the knees flexed, and the bladder emptied [8]. Fetal head position and rotation were assessed qualitatively in real time and used for clinical decision‐making, including assessment of whether the degree of rotation was < 45° or ≥ 45°.

The MA was measured in the axial plane using a transperineal approach. It was defined as the angle between the fetal cranial midline, identified as the falx cerebri or thalamic symmetry line, and the maternal anteroposterior pelvic axis, as previously described [9]. The AoP was measured in the parasagittal plane as the angle between the longitudinal axis of the pubic symphysis and the tangent line from the inferior edge of the pubic symphysis to the fetal head contour [13]. Both measurements were obtained from stored still images and analyzed offline by two trained obstetricians blinded to the neonatal outcomes. Discrepant measurements were reviewed jointly, and the final value was determined by consensus.

### 2.3 Outcome Definition

The primary outcome was the presence of forceps compression marks over the neonatal periorbital region, as identified by neonatologists after forceps delivery. All affected neonates underwent ophthalmologic evaluation and were classified as the “compression mark group.” Periorbital compression marks were regarded as a surrogate indicator of mechanical compression by the forceps blade and were not considered evidence of ophthalmic injury. All neonates in the compression mark group underwent ophthalmologic examination within the first 3 days after delivery. Ophthalmologist‐confirmed findings, including retinal hemorrhage, subconjunctival hemorrhage, corneal epithelial injury, hyphema, and oculomotor dysfunction, were recorded as secondary descriptive outcomes.

### 2.4 Statistical Analysis

Continuous variables were presented as medians with interquartile ranges (IQRs) and were compared using the Mann–Whitney *U* test. Receiver operating characteristic (ROC) curve analysis was performed to evaluate the predictive performance of MA for forceps‐related compression over the neonatal periorbital region. The optimal cutoff value was determined using Youden's index. Statistical significance was set at *p* < 0.05. All analyses were performed using JMP Pro, version 17 (SAS Institute Inc., Tokyo, Japan).

## 3 Results

A total of 166 term singleton forceps deliveries were included in the final analysis, comprising 67 neonates in the compression mark group and 99 in the control group. Both the compression mark and control groups included eligible forceps deliveries performed between January 2019 and December 2022.

Nine neonates in the compression mark group had ophthalmic findings. All ophthalmic findings occurred ipsilateral to the forceps compression marks; no contralateral ophthalmic findings were observed. These included retinal hemorrhage in one neonate, corneal epithelial injury in two, and subconjunctival hemorrhage in six. The median MA in this cohort was 25.0° (IQR, 21°–35°). All findings resolved spontaneously with observation alone (Table 1).

**TABLE 1:** Ophthalmic findings and midline angle (MA) of neonates who underwent ophthalmologic evaluation after forceps delivery in the compression mark group.

Case number	Ophthalmic findings	MA (degrees)
1	Retinal hemorrhage	40
2	Corneal epithelial injury	20
3	Corneal epithelial injury	21
4	Subconjunctival hemorrhage	37
5	Subconjunctival hemorrhage	25
6	Subconjunctival hemorrhage	11
7	Subconjunctival hemorrhage	35
8	Subconjunctival hemorrhage	26
9	Subconjunctival hemorrhage	22

No significant between‐group differences were observed in maternal age, height, weight, neonatal head circumference, or AoP. However, the median MA was significantly greater in the compression mark group than in the control group (22.0° [IQR, 13.0°–30.0°] vs. 13.0° [IQR, 1.0°–18.5°], *p* < 0.001) (Table 2). ROC curve analysis was performed to assess the predictive value of MA for the presence of forceps compression marks in the neonatal periorbital region. The area under the curve (AUC) was 0.732 (95% CI, 0.649–0.807), indicating moderate discrimination. Based on Youden's index, the optimal cutoff value for MA was determined to be 22°, yielding a sensitivity of 47.8% and specificity of 90.9%. (Figure 1).

**TABLE 2:** Comparison of maternal and neonatal demographics and ultrasound findings.

	Compression mark group (*n* = 67)	Control (*n* = 99)	*p*
Maternal age (years)	35.0 (IQR: 33–38)	36.0 (IQR: 33–39)	0.46
Maternal height (cm)	158.0 (IQR: 154.0–162.0)	159.0 (IQR: 155.0–162.5)	0.14
Neonatal weight (g)	3148 (IQR: 2826–3385)	3056 (IQR: 2882–3335)	0.76
Neonatal HC (cm)	33.8 (IQR: 33.0–35.0)	34.0 (IQR: 33.0–35.0)	0.55
AoP (degrees)	168 (IQR: 162.5–171.0)	166 (IQR: 160.0–172.5)	0.14
MA (degrees)	22.0 (IQR: 13.0–30.0)	13.0 (IQR: 1.0–18.5)	< 0.001

Abbreviations: AoP, angle of progression; HC, head circumference; IQR, interquartile range; MA, midline angle.

**FIGURE 1:**
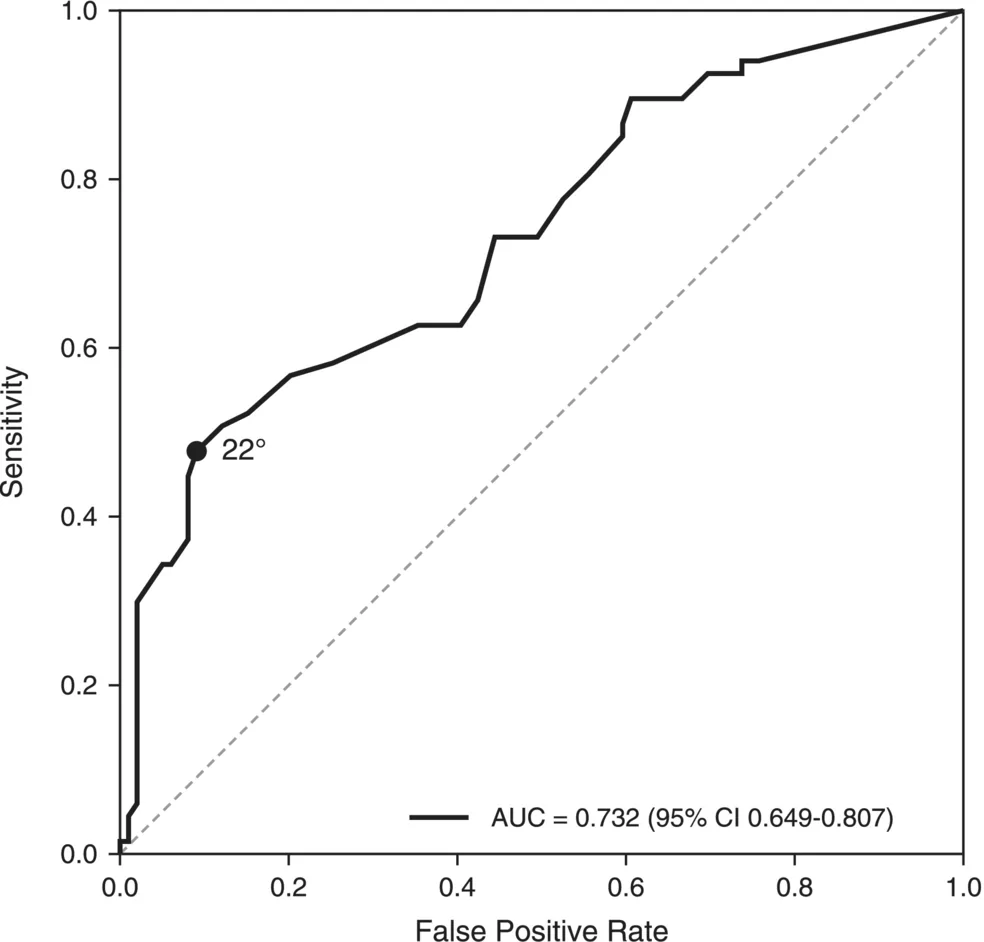
Receiver operating characteristic curve for midline angle predicting neonatal periorbital compression marks after forceps delivery. The area under the curve was 0.732 (95% CI, 0.649–0.807). The optimal cutoff value determined by Youden's index was 22°, yielding a sensitivity of 47.8% and specificity of 90.9%.

## 4 Discussion

An increased MA was significantly associated with the incidence of neonatal periorbital compression marks after forceps delivery. Specifically, an MA threshold of 22° yielded a specificity of 90.9% for identifying neonates with clinical signs of orbital compression. These findings suggest that an MA greater than 22° may be associated with an increased risk of neonatal periorbital compression during forceps delivery.

Although malrotation is a well‐recognized risk factor for neonatal periorbital compression during forceps delivery, our findings suggest that MA assessment may improve risk stratification even among fetuses with occiput anterior presentation, which has traditionally been regarded as a low‐risk presentation for neonatal periorbital compression. Moreover, although some experts recommend routine sonographic confirmation of the fetal head position before attempting operative vaginal delivery, these recommendations do not address MA assessment in relation to neonatal safety [8]. To our knowledge, this is the first study to evaluate the association between MA and the risk of neonatal periorbital compression. Previous studies have demonstrated that higher MA values are associated with prolonged second‐stage labor and higher rates of operative delivery, including forceps and vacuum deliveries [14, 15], whereas our findings extend the clinical relevance of MA by highlighting its potential role in risk stratification for forceps‐related neonatal periorbital compression.

Clinically, MA may provide supplementary information when assessing the likelihood of neonatal periorbital compression marks before forceps delivery. Our institutional policy permits forceps delivery when the MA is < 45°; however, this institutional criterion is distinct from the exploratory value of > 22° identified in the present ROC analysis [16]. Although an MA > 22° showed relatively high specificity (90.9%), its limited sensitivity (47.8%) indicates that more than half of neonates with periorbital compression marks would not be identified using this value. Therefore, for risk stratification of neonatal periorbital compression marks, MA should be interpreted alongside fetal head position, station, the indication for operative delivery, and operator experience, rather than used in isolation.

This study has some limitations. First, its retrospective design precluded causal inference. Periorbital compression marks may be influenced by procedural factors, including operator experience, fetal station, the number and duration of traction attempts, difficulty of forceps application, and blade repositioning. These variables were not consistently available and therefore could not be evaluated. Therefore, residual confounding by unmeasured procedural factors cannot be excluded, and the observed association between MA and periorbital compression marks should not be interpreted as demonstrating an independent or causal effect of MA. Second, periorbital compression marks represent a surrogate outcome and do not necessarily indicate clinically significant orbital injury. However, because clinically apparent ocular injury is rare and usually accompanies compression marks, this endpoint provides a practical surrogate for evaluating procedural safety. Finally, because one case with an MA of 11° (Case 6) was observed, a low MA does not completely exclude the possibility of periorbital compression. Neonatal periorbital compression may occur when the forceps blade slips over the orbital rim and compresses the orbital region during traction [17]. Therefore, clinicians should not rely solely on MA values for risk assessment, and meticulous forceps technique remains essential to minimize the risk of neonatal injury.

In conclusion, a MA greater than 22° on transperineal ultrasonography was associated with an increased risk of neonatal periorbital compression during forceps delivery in occiput anterior presentations. Assessment of MA before forceps application may improve risk stratification and enhance procedural safety during forceps delivery.

## Author Contributions

**Seisuke Sayama:** conceptualization, methodology, investigation, validation, project administration, resources, writing – original draft, writing – review and editing, software, formal analysis. **Takahiro Seyama:** conceptualization, methodology, data curation, software, formal analysis, investigation, validation, writing – review and editing, resources. **Takayuki Iriyama:** investigation, writing – review and editing, supervision. **Eriko Yano:** resources, writing – review and editing. **Haruka Matsui:** resources, writing – review and editing. **Kensuke Suzuki:** resources, writing – review and editing. **Mari Ichinose:** resources, writing – review and editing. **Masatake Toshimitsu:** resources, writing – review and editing. **Keiichi Kumasawa:** resources, writing – review and editing. **Miyuki Harada:** supervision, writing – review and editing. **Yasushi Hirota:** writing – review and editing, supervision.

## Funding

The authors have nothing to report.

## Disclosure

This study was presented at the 62nd Annual Meeting of the Japan Society of Perinatal and Neonatal Medicine, held in Yokohama, Japan, on July 14, 2026.

## Ethics Statement

The study protocol was approved by the Institutional Review Board of the University of Tokyo (Approval No. 3053). The requirement for informed consent was waived because of the retrospective nature of the study, and an opt‐out procedure was provided in accordance with institutional policy.

## Consent

The requirement for written informed consent was waived because of the retrospective nature of the study and the use of anonymized clinical data. An opt‐out procedure was provided through the hospital website.

## Conflicts of Interest

The authors declare no conflicts of interest.

## Data Availability

The data supporting the findings of this study are not publicly available because public deposition of patient‐level clinical data were not included in the study protocol approved by the Institutional Review Board of the University of Tokyo. Public disclosure of these data would therefore be inconsistent with the approved ethical framework and could compromise participant confidentiality.
